# Absence, loss-of-function, or inhibition of *Escherichia coli* AcrB does not increase expression of other efflux pump genes supporting the discovery of AcrB inhibitors as antibiotic adjuvants

**DOI:** 10.1093/jac/dkab452

**Published:** 2021-12-13

**Authors:** Maria Laura Ciusa, Robert L Marshall, Vito Ricci, Jack W Stone, Laura J V Piddock

**Affiliations:** Institute of Microbiology and Infection, College of Medical and Dental Sciences, University of Birmingham, Birmingham, UK

## Abstract

**Objectives:**

To determine whether expression of efflux pumps and antibiotic susceptibility are altered in *Escherichia coli* in response to efflux inhibition.

**Methods:**

The promoter regions of nine efflux pump genes (*acrAB*, *acrD, acrEF, emrAB, macAB, cusCFBA, mdtK, mdtABC, mdfA*) were fused to *gfp* in pMW82 and fluorescence from each reporter construct was used as a measure of the transcriptional response to conditions in which AcrB was inhibited, absent or made non-functional. Expression was also determined by RT-qPCR. Drug susceptibility of efflux pump mutants with missense mutations known or predicted to cause loss of function of the encoded efflux pump was investigated.

**Results:**

Data from the GFP reporter constructs revealed that no increased expression of the tested efflux pump genes was observed when AcrB was absent, made non-functional, or inhibited by an efflux pump inhibitor/competitive substrate, such as PAβN or chlorpromazine. This was confirmed by RT-qPCR for PAβN and chlorpromazine; however, a small but significant increase in *macB* gene expression was seen when *acrB* is deleted. Efflux inhibitors only synergized with antibiotics in the presence of a functional AcrB. When AcrB was absent or non-functional, there was no impact on MICs when other efflux pumps were also made non-functional.

**Conclusions:**

Absence, loss-of-function, or inhibition of *E. coli* AcrB did not significantly increase expression of other efflux pump genes, which suggests there is no compensatory mechanism to overcome efflux inhibition and supports the discovery of inhibitors of AcrB as antibiotic adjuvants.

## Introduction

The contribution of multidrug efflux to antibiotic resistance is well established and in Gram-negative bacteria the resistance nodulation-division (RND) family of transporters are reported to have the greatest effect on antibiotic susceptibility.^1^*Escherichia coli* has seven characterized RND transporters, of which AcrB, AcrD, AcrF, MdtB, MdtC and MdtF belong to the hydrophobic/amphiphilic efflux (HAE) subfamily, and CusA to the heavy metal efflux (HME) subfamily.^2^ A further 30 putative efflux pumps have been identified in *E. coli* with some belonging to transporter families other than RND.^3^

Overexpression of chromosomally encoded efflux pumps has been described as a mechanism of drug resistance in clinical and environmental isolates of multiple Gram-negative species.^4–8^ For this reason, MDR efflux systems are attractive targets for antimicrobial drug discovery as addition of efflux inhibitors to currently available antibiotics will extend their spectrum of activity and lengthen the period of effective use. However, the bacterial response to efflux inhibition and drug–inhibitor combinations still needs to be investigated.

Regulation of efflux pumps is complex and involves local as well as global regulators. The AcrAB-TolC system is constitutively expressed and altering the expression of the AcrB transporter has a profound effect on susceptibility to a wide variety of compounds.^9^ Most other efflux pumps are expressed at low levels under normal conditions or are synthesized *de novo* in response to a specific environmental stress.^10^ Nonetheless, overexpression of other pumps has been shown to confer a decrease in susceptibility to several toxic compounds.^3^ Several studies have shown that in the presence of AcrB, deletion of efflux genes has little to no impact on antibiotic susceptibility.^11–14^ High-level expression of efflux pumps can be achieved by mutation in the elements regulating their expression, or by the presence of their inducers.^15–19^ Increased expression of other efflux pumps in the absence of AcrB (or its homologue in other Gram-negative bacteria e.g. MexB) is hypothesized to be a compensatory effect due to the absence of the main RND efflux transporter gene of the species.^4–8^^,^^15^^,^^17^^,^^19–22^ However, in *Salmonella enterica* serovar Typhimurium this compensatory effect is not seen when AcrB is present but functionally inactivated.^23^

In this study, we sought to determine whether chemical inhibition of AcrB impacts the expression of four RND (*acrD, acrEF, mdtABC, cusCFBA*), two MFS (*emrAB, mdfA*) one ABC (*macAB*) and one MATE (*mdtK*) efflux pump genes and drug susceptibility in *E. coli*. These pump genes were chosen as other studies indicated that, of 37 putative efflux pump-encoding open reading frames, these eight may influence susceptibility or tolerance to antibiotics and other toxic compounds.^2^^,^^3^^,^^5^^,^^11^^,^^12^^,^^14^ Two chemical inhibitors, phenylalanine-arginine-β-naphthylamide (PAβN), a well-studied efflux inhibitor, and chlorpromazine (CPZ), a phenothiazine compound, and an AcrAB competitive substrate^24–26^ were investigated.

## Materials and methods

### Bacterial strains and plasmids

All strains and plasmids used in this study are shown in Table 1. The method of Datsenko and Wanner^27^ was used to remove *acrB* from the chromosome of MG1655, with the *acrB::aph* cassette amplified from the Keio collection strain JW0451 using primers *acrB*_upst_F (5′-GGTGTCCAGGTAAAAGCACAAG-3′), and *acrB*_dnst_R (5′-GACGTAATAACCGAGGAATGAATAAAG-3′); subsequent removal of the selection marker was also carried out as described by Datsenko and Wanner.^27^ The method of Kim *et al.*^28^ was used for construction of chromosomal missense mutations; the inserts were synthesized by GenScript, PCR-amplified from the supplied plasmids using Q5 polymerase (NEB) (primers listed in Table S1, available as Supplementary data at *JAC* Online) and purified using a gel extraction kit (Neo BioTech) according to the manufacturer’s instructions. For each experiment using these missense mutants, the presence of the mutation was confirmed by a PCR check from colony lysates using the primers listed in Table S2. This was a necessary precaution as reversion to the wild-type sequence has been observed for an AcrB-inactivating mutation.^24^ Each forward primer has been used with the corresponding int-check reverse primer to selectively amplify only if the template has the WT or the mutated sequence. Cycling conditions were 95°C for 3 min, followed by 30 cycles of 95°C for 10 s and 72°C for 30 s (two-step).

**Table 1.** Bacterial strains and plasmids used in this study

Strain or plasmid	Description	Source or reference
MG1655	*Escherichia coli* K-12 derivative	
MG1655 *ΔacrB*	MG1655 lacking the *acrB* gene	This study
MG1655 AcrB(D408A)	MG1655 with a missense mutation that inactivates AcrB	^32^
MG1655 AcrD(D408A)	MG1655 with a missense mutation that inactivates AcrD	This study
MG1655 AcrF(D408A)	MG1655 with a missense mutation that inactivates AcrF	This study
MG1655 MacB(K47L)	MG1655 with a missense mutation that inactivates MacB^13^	This study
MG1655 MdtK(D368A)	MG1655 with a missense mutation predicted to inactivate MdtK	This study
MG1655AcrB(D408A)/AcrD(D408A)	AcrB(D408A) missense mutation introduced to MG1655 AcrD(D408A)	This study
MG1655AcrB(D408A)/AcrF(D408A)	AcrB(D408A) missense mutation introduced to MG1655 AcrF(D408A)	This study
MG1655 AcrB(D408A)/MacB(K47L)	AcrB(D408A) missense mutation introduced to MG1655 MacB(K47L)	This study
MG1655AcrB(D408A)/MdtK(D368A)	AcrB(D408A) missense mutation introduced to MG1655 MdtK(D408A)	This study
pMW82	Plasmid without a promoter from which to express the encoded *gfp*	^29^
pMW82-*acrABp*	pMW82 with the *acrA* promoter sequence regulating expression of *gfp*	This study
pMW82-*acrDp*	pMW82 with the *acrD* promoter sequence regulating expression of *gfp*	This study
pMW82*-acrEFp*	pMW82 with the *acrE* promoter sequence regulating expression of *gfp*	This study
pMW82-*cusCFBAp*	pMW82 with the *cusC* promoter sequence regulating expression of *gfp*	This study
pMW82-*emrABp*	pMW82 with a predicted *emrA* promoter sequence regulating expression of *gfp*	This study
pMW82-*macABp*	pMW82 with the *macA* promoter sequence regulating expression of *gfp*	This study
pMW82-*mdfAp*	pMW82 with a predicted *mdfA* promoter sequence regulating expression of *gfp*	This study
pMW82-*mdtABCp*	pMW82 with the *mdtA* promoter sequence regulating expression of *gfp*	This study
pMW82-*mdtKp*	pMW82 with a predicted *mdtK* promoter sequence regulating expression of *gfp*	This study

Reporter plasmids were constructed by amplifying the known or predicted regulatory region of the genes encoding nine *E. coli* efflux pumps (AcrAB, AcrD, AcrEF, MdtABC, EmrAB, MacAB, CusCFBA, MdtK, MdfA) using Q5 polymerase and the primers shown in Table S3, purified by gel extraction and cloned into the BamHI and XbaI restriction sites of pMW82.^29^ Each efflux pump gene promoter–GFP fusion was transformed into *E. coli* MG1655, and its mutants, MG1655 *acrB* D408A and MG1655 Δ*acrB*. Empty vector control strains were constructed by transforming the promoterless pMW82 plasmid into the same three strains. Bacterial strains were grown overnight at 37°C in Luria–Bertani broth, Lennox formulation (Sigma). PAβN was supplied by Cambridge Bioscience, antibiotics and other toxic compounds including chlorpromazine were supplied by Sigma–Aldrich.

### Gene expression analysis by GFP reporter assays

To confirm that the reporters responded to efflux promoter activity, their basal expression in minimal medium was measured, and positive control compounds and conditions were sought (Table S4).

To determine how the expression of efflux pump genes in this study changes in response to chemical inhibition of AcrB, 10 μL of chlorpromazine or PAβN, at 10× final concentration were added to black-sided, clear flat-bottomed 96-well plates (Greiner). Overnight cultures were diluted in MOPS minimal medium (Teknova) supplemented with glucose, 400 mg/L histidine and 1 mg/L thiamine, incubated at 37°C until OD_600_ of approximately 0.6 was reached and 90 μL per well used to inoculate assay plates. GFP fluorescence (excitation 492 nm, emission 520 nm) and growth kinetics (absorbance at OD_600_) were measured every 3 min for 5 h on a FLUOstar Omega plate reader (BMG Labtech). To determine how efflux pumps in this study respond at the transcriptional level when AcrB is absent or made non-functional, overnight cultures were diluted 1/1000 and 100 μL per well used to inoculate assay plates. GFP fluorescence and OD_600_ were measured every 3 min for 15 h on a FLUOstar Omega plate reader (BMG Labtech). Two biological and three technical replicates of each culture were used in each assay. The blank corrected fluorescence value was divided by the OD_600_ value to give specific fluorescence (units of fluorescence per unit OD). The maximum specific fluorescence at any timepoint was used to compare the effect of each condition on each reporter.

### Gene expression analysis by qPCR

The RT-qPCR assay was used for: (i) confirming the functionality of the GFP reporters by measuring *gfp* expression, using primers listed in Table S5; and (ii) testing efflux pump genes expression from the chromosome, in the presence of chlorpromazine or PAβN in MG1655 and its *acrB* deletion and AcrB loss-of-function mutants, by RT-qPCR assay using primers listed in Table 2.

**Table 2.** Primers for qPCR on efflux pump genes

Primer name	Sequence (5′–3′)	Tm (°C)	Amplicon length (bp)
*acrB qPCR Fw*	AAGAAGCTACCCGTAAGTCG	57.3	107
*acrB qPCR Rv*	AGTAGAACCGCCAAAGAAGG	57.3	
*acrD qPCR Fw*	TGGAATCGTTAGTGAAGCAG	56.3	138
*acrD qPCR Rv*	CAGCCAGACACAGGAATAC	56.7	
*acrF qPCR Fw*	AGGAACGCTTATCGGGAAAC	57.3	130
*acrF qPCR Rv*	CCTAACGGCACTACCAACATA	57.9	
*emrB qPCR Fw*	TCTCATTGGCGGAAATAATCAG	57.9	132
*emrB qPCR Rv*	TAAACCCGTTCAACCCGAAT	56.5	
*cusA qPCR Fw*	TGAAGAGAGTTCTGCGTCTG	57.3	152
*cusA qPCR Rv*	GCACAATGGCATACTGATACTC	58.4	
*macB qPCR Fw*	GGCGTCTTGAAAACTGTTGA	55.2	106
*macB qPCR Rv*	GTCACTGACACCAGCATAATA	55.9	
*mdfA qPCR Fw*	TTGATTGGGTTCCTACTTCG	55.3	141
*mdfA qPCR Rv*	CCAGACAGGTGACGATAAAC	57.3	
*mdtB qPCR Fw*	ATGGACACCGAAAAGACGCT	57.3	142
*mdtB qPCR Rv*	TGCCGAGCACGATATACATC	57.3	
*mdtC qPCR Fw*	ATCCCGAAAACCTTCTTCCC	57.3	108
*mdtC qPCR Rv*	GAAATCCTGCAACTTACCGC	57.3	
*mdtK qPCR Fw*	TAATGTTCGTGCTTCCAATG	55.2	128
*mdtK qPCR Rv*	CATACAGACACCCACCATAA	55.3	
16S rRNA qPCR Fw	GCTAATACCGCATAACGTCG	57.3	139
16S rRNA qPCR Rv	TCATCCTCTCAGACCAGCTA	57.3	

Primers were designed based on the sequence for the *E. coli* K-12 complete genome (accession number NC_000913).

Three cultures of each strain were cultured at 37°C in the appropriate medium until an OD_600_ of approximately 0.6 was reached. RNA was extracted, cDNA prepared, and qPCR performed and analysed as previously described,^23^ using 16S rRNA and *gyrB* as the reference housekeeping genes for calculation of relative expression. Primers used are listed in Table S5. Real time qPCR was carried out in a CFX96 real-time machine (Bio-Rad, UK) using the following cycling conditions: 95°C for 3 min, followed by 40 cycles of 95°C for 10 s, 57.3°C for 10 s and 72°C for 10 s. Data were analysed using CFX Manager (Bio-Rad, UK) and expression ratios were calculated using the ΔΔCt method and normalized to the expression of 16S rRNA.

### Antimicrobial susceptibility

Susceptibility of each strain to each compound was tested using the method recommended by EUCAST. EUCAST guidelines were followed conforming to ISO 20776–1:2006.^30^^,^^31^ Antibiotics and efflux pump inhibitors (EPIs) were made up according to the manufacturer's instructions. *E. coli* ATCC 25922 was used as the control strain.

### Statistical analysis

Statistically significant differences in GFP reporter assays were identified with the Student’s *t*-test comparing the maximum fluorescence value achieved in a specific condition with the fluorescence value achieved in absence of that condition with values of *P < *0.05 indicating significance.

Statistically significant differences in RT–qPCR assays were identified with the pairwise *t*-test comparing the expression in three conditions (no EPI, chlorpromazine 16 mg/L, and PAβN 32 mg/L) and in three genetic backgrounds (MG1655 WT, MG1655 *acrB* D408A mutant, and *acrB* deletion mutant) with values of *P < *0.05 indicating significance. Expression of each gene was tested with a separate pairwise *t*-test, to analyse the chemical and genetic conditions both alone and in combination.

## Results

### Except for AcrB, loss of function mutations in efflux pump genes had no effect on antimicrobial susceptibility

In single efflux pump gene deletion mutants, only *acrB* had an effect upon antibiotic susceptibility.^3^ In previous publications, the *acrB* D408A missense mutation, causing AcrB to be expressed at normal levels but functionally inactivated, conferred increased susceptibility to AcrB substrates and had a different transcriptional response to deletion of the same gene.^23^^,^^32^ In this study, single missense mutants were made in five other efflux pump genes in *E. coli* to investigate the hypothesis that bacteria with pump gene deletions may be responding to loss of a large membrane protein and not loss of efflux. The susceptibility of these mutants with missense mutations known or predicted to cause loss of function of the encoded efflux pump was determined for known efflux pump substrates. Only mutational inactivation of AcrB conferred a change in susceptibility in *E. coli* MG1655. To determine if the presence of AcrB masked the effect of loss of another efflux pump gene, double mutants were also constructed with loss of function of *acrB* and another efflux pump gene. However, susceptibility of the double mutants to antibiotics was the same as for the strain with only the mutation in *acrB*; mutation in the other efflux pumps caused no further effect (Table 3).

**Table 3.** Susceptibility of *E. coli* strains with mutational inactivation of one or two efflux pumps against a selection of antibiotics and toxic agents

	MIC (mg/L)													
MG1655 Genotype	CIP	NAL	KAN	CHL	TET	ERY	THZ	TFP	CPZ	AMI	DCA	SDS	EtBr	CCCP
WT	0.016	8	1	8	2	64	512	512	128	256	>4096	>1024	512	64
AcrB D408A	**<0.008**	**2**	0.5	**1**	**0.5**	**8**	**32**	**32**	**32**	**64**	**128**	**256**	**16**	32
AcrD D408A	0.015	8	0.5	8	2	64	512	512	128	256	>4096	>1024	512	64
AcrF D408A	0.015	8	1	8	2	64	512	512	128	256	>4096	>1024	512	64
MacB K47L	0.015	4	1	8	2	64	512	512	128	256	>4096	>1024	512	32
MdtK D368A	0.015	8	1	8	2	64	512	512	128	256	>4096	>1024	512	64
AcrD D408A/AcrB D408A	**<0.008**	**2**	0.5	**1**	**0.5**	**8**	**32**	**32**	**32**	**64**	**128**	**256**	**16**	32
AcrF D408A/AcrB D408A	**<0.008**	**2**	0.5	**1**	**0.5**	**8**	**32**	**32**	**32**	**64**	**128**	**256**	**16**	32
MacB K47L/AcrB D408A	**<0.008**	**2**	0.5	**1**	**0.5**	**8**	**32**	**32**	**32**	**64**	**256**	**256**	**16**	32
MdtK D368A/AcrB D408A	**<0.008**	**2**	0.5	**1**	**0.5**	**8**	**32**	**32**	**32**	**64**	**256**	**256**	**16**	32

Values shown in bold are significantly decreased compared with the wild-type strain. CIP, ciprofloxacin; NAL, nalidixic acid; KAN, kanamycin; CHL, chloramphenicol; TET, tetracycline; ERY, erythromycin; THZ, thioridazine; TFP, trifluoperazine; CPZ, chlorpromazine; AMI, amitryptiline; DCA, deoxycholic acid; SDS, sodium dodecyl sulfate; EtBr, ethidium bromide; CCCP, carbonyl cyanide *m*-chlorophenyl hydrazine.

### Chemical inhibition of efflux only influenced the susceptibility profile in the presence of a functional AcrB

To further test whether in *E. coli* there is a contribution of efflux pumps other than AcrB in antibiotic susceptibility, the MICs of a selection of antibiotics and other toxic compounds were determined with wild-type MG1655, MG1655 *ΔacrB* and the MG1655 strain producing the inactive D408A variant of AcrB alone and in the presence of 10 mg/L of efflux inhibitor (chlorpromazine and PAβN) (Table 4). In the WT, the presence of chlorpromazine did not show any effect on MIC values, while the presence of PAβN significantly decreased the MICs of chloramphenicol, erythromycin and novobiocin, which are substrates of AcrB. Except for the combination of PAβN with erythromycin (which reduced the erythromycin MIC from 8 to 1 mg/L in both the AcrB-deleted and functionally inactivated mutants), the antimicrobial activity of the tested antibiotics was unaffected by either chlorpromazine or PAβN in these mutants.

**Table 4.** Susceptibility of *E. coli* MG1655 WT and AcrB mutants against a selection of agents, with and without the presence of 10 mg/L PAβN or chlorpromazine (CPZ)

Inhibitor	AcrB variant	MIC (mg/L)								
		CIP	NOR	TET	CHL	ERY	NOV	KAN	EtBr	ACR
None	WT	0.015	0.06	1	8	64	256	1	512	64
	deleted	0.004	0.03	1	1	8	4	1	8	8
	D408A	0.004	0.03	0.5	1	8	4	1	8	8
PAβN	WT	0.015	0.12	1	**1**	**8**	**64**	2	512	128
	deleted	0.004	0.03	0.25	1	*1*	2	1	8	8
	D408A	0.004	0.03	0.25	1	*1*	2	1	4	4
CPZ	WT	0.015	0.12	1	4	64	256	2	256	128
	deleted	0.004	0.03	0.25	1	8	4	2	2	8
	D408A	0.004	0.03	0.25	1	8	4	2	2	8

Bold font indicates statistically significant decreased value compared with in the absence of efflux inhibitor. Italic font indicates statistically significant decreased value compared to the wild-type strain in the same testing condition. CIP, ciprofloxacin; NOR, norfloxacin; TET, tetracycline; CHL, chloramphenicol; ERY, erythromycin; NOV, novobiocin; KAN, kanamycin; EtBr, ethidium bromide; ACR, acriflavine.

### Reporter construct functionality was confirmed by RT-qPCR

**Figure 1.**
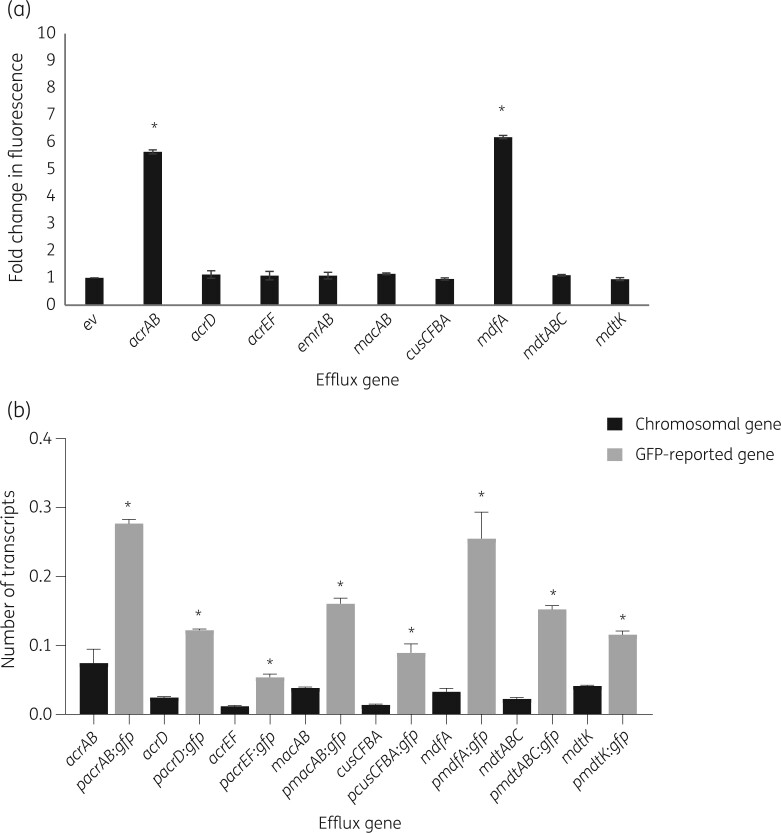
Basal expression from transcriptional GFP reporters. (a) Fold change in GFP fluorescence in *E. coli* MG1655 in minimal medium. Values are averages of two biological and three technical replicates for each reporter strain. Maximum specific fluorescence, where present, was shown at OD_600nm_ 0.6. Student’s *t-*test was performed comparing the maximum fluorescence value achieved by each reporter with the fluorescence value of the empty vector (ev), promoter-less pMW82, with values of *P *< 0.05 indicating significance. An asterisk indicates a statistically significantly increased fluorescence compared with empty vector background fluorescence. ev, empty vector. (b) Relative number of transcripts of efflux pump genes in *E. coli* MG1655 in minimal medium measured from the chromosome and as *gfp* reported expression. Values are averages of three biological and two technical replicates for each strain and condition. Gene transcription was measured at growth corresponding to OD_600nm_ 0.6. An asterisk indicates a statistically significant (*P < *0.05) increased expression compared with chromosomal expression.

GFP expression was measured for nine efflux pump gene transcriptional reporter plasmids (Table 2). The basal level of GFP expression was measured by a fluorescence assay and by RT-qPCR for each reporter in *E. coli* MG1655 during growth in minimal medium (MOPS). The basal level of fluorescence was very low for seven of the nine reporters with only pMW82*acrAB*p and pMW82*mdfA*p showing fluorescence values significantly higher (*P < *0.05) than those obtained with the corresponding empty vector (Figure 1a).

When we measured GFP fluorescence in conditions able to cause efflux pump induction or compensatory overexpression (Table S4), only three reporters responded: pMW82*acrAB*p, pMW82*acrEF*p and pMW82*cusCFBA*p (Figure S1 and Figure S2). RT-qPCR analysis of the reporters confirmed they were all functional and expressing the reported genes at a higher level than the basal expression from the chromosome (Figure 1b and Table S6).

### Except for acrB, basal expression was low for the other eight efflux pump genes tested

**Figure 2.**
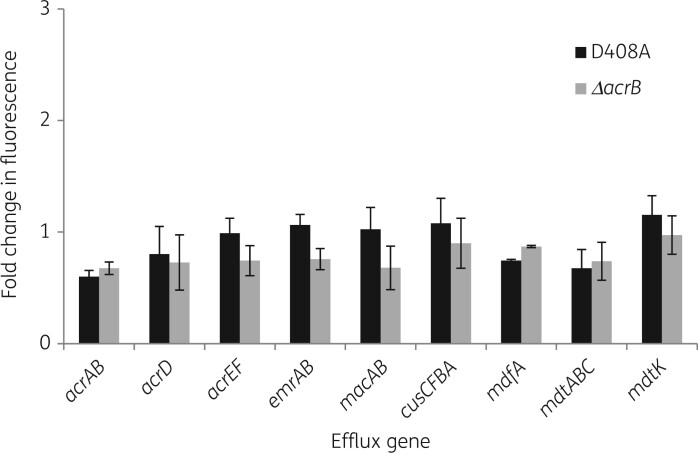
Expression from transcriptional GFP reporters in *acrB* mutant backgrounds. Fold change in GFP fluorescence expressed from efflux pump gene promoter reporter constructs in *E. coli* MG1655 *acrB* D408 and Δ*acrB* mutant compared with expression in WT strain. Values are averages of three biological and two technical replicates for each reporter strain. Maximum specific fluorescence was seen at OD_600nm_ 0.6. Student’s *t*-test was performed comparing the maximum fluorescence value achieved in *acrB* mutants with the fluorescence value of the corresponding WT strain, with values of *P *< 0.05 indicating significance.

GFP expression from the reporter constructs was tested in MG1655 in which *acrB* had been deleted and a strain in which *acrB* had been mutated to give AcrB D408A. Loss of function or functional inactivation by missense mutation of *acrB* did not cause any statistically significant increase in GFP fluorescence compared with WT *acrB* (Figure 2).

Data from RT-qPCR showed that the *acrB* gene (where present) was expressed 2 to 6-fold more than the other efflux genes (up to 10-fold in the case of the CusCFBA pump) confirming the overall low expression of the other efflux pumps shown in the GFP reporting assay in the three genetic backgrounds (MG1655 WT, MG1655 *acrB* D408A mutant, *acrB* deletion mutant) (Table 5).

**Table 5.** Relative expression of efflux pump genes in the presence of chlorpromazine or PAβN

	Copies of transcript per copy of 16S rRNA								
	WT			*acrB* D408A			*ΔacrB*		
Gene	No EPI	CPZ	PAβN	No EPI	CPZ	PAβN	No EPI	CPZ	PAβN
*acrB*	0.10	0.10	0.11	0.10	0.09	0.11	0.00	0.00	0.00
*acrD*	0.05	0.04	0.04	0.04	0.03	0.04	0.05	0.04	0.04
*acrF*	0.03	0.02	0.02	0.01	0.01	0.01	0.04	0.03	0.03
*emrB*	0.06	0.05	0.05	0.03	0.03	0.03	0.01	0.01	0.01
*cusBA*	0.00	0.00	0.00	0.00	0.00	0.00	0.00	0.00	0.00
*macB*	0.02	0.02	0.03	0.01	0.01	0.02	**0.05**	0.04	0.05
*mdfA*	0.02	0.02	0.01	0.03	0.02	0.02	0.02	0.02	0.01
*mdtB*	0.05	0.04	0.04	0.02	0.02	0.02	0.02	0.02	0.02
*mdtC*	0.05	0.05	0.04	0.02	0.02	0.03	0.01	0.01	0.02
*mdtK*	0.05	0.04	0.04	0.04	0.03	0.04	0.04	0.03	0.04

Values are averages of three biological and two technical replicates for each strain and condition. Gene transcription was measured at growth corresponding to OD_600nm_ 0.6. For each of the genes, a pairwise *t*-test was performed for comparing the expression in the three conditions [no EPI, CPZ (16 mg/L) and PAβN (32 mg/L)] and in the three genetic backgrounds (MG1655 WT, MG1655 *acrB* D408A mutant, *acrB* deletion mutant) with values of *P < *0.05 indicating significance. No significant differences were found in presence of EPIs. Bold font indicates statistically significantly increased expression of *acrB* mutant background versus WT. EPI, efflux inhibitor; CPZ, chlorpromazine; PAβN, phenyl-arginine-β-naphthylamide.

Similar to the GFP fluorescence assay, data obtained from RT-qPCR showed that efflux pump expression was overall the same or even decreased when AcrB was absent or not functional, with the only exception being *macB* expression in the *acrB* deletion mutant, which was significantly increased 2.6-fold (Table 5). Expression of the *acrF* gene was also increased (1.6-fold) in the *acrB* deletion mutant and the *mdfA* gene showed a 1.4-fold increase in expression in the *acrB* D408A mutant, but those were not statistically significant, having *P* values* > *0.05 (Table 5).

### Inhibition of AcrB has no significant effect on expression of efflux pump genes

**Figure 3.**
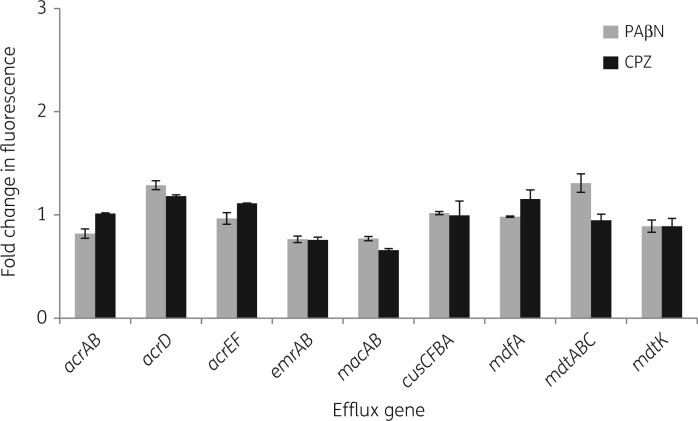
Expression from transcriptional GFP reporters in the presence of efflux inhibitors. Fold change in GFP fluorescence in MG1655 in the presence of 32 mg/L PAβN or 16 mg/L CPZ compared with the no-compound condition. Values are averages of two biological and three technical replicates for each reporter strain. Maximum specific fluorescence was seen at OD_600nm_ 0.6. Student’s *t*-test was performed comparing the maximum fluorescence value achieved in the presence of compound with the fluorescence value of the culture in its absence, with values of *P *< 0.05 indicating significance.

In the presence of sub-inhibitory concentrations (1/8 MIC) of PAβN (32 mg/L) or chlorpromazine (16 mg/L), there was no increase in GFP expression from the reporter constructs compared with expression in the absence of EPI (Figure 3). The basal expression levels from efflux pump gene promoters were very low, and the subsequent amount of GFP produced provided fluorescence levels below the limit of detection of the GFP fluorescence assay for some of the constructs. Therefore, expression of the efflux pump genes in the presence of sub-inhibitory concentrations of chlorpromazine and PAβN was also quantified by RT-qPCR assay in the three background strains and compared with the expression of those genes in the absence of efflux inhibitor. No significant differences in expression were found in the presence of either of the compounds (Table 5).

## Discussion

Overcoming bacterial multidrug resistance by inhibiting efflux of antibiotics from the bacterial cell is an attractive prospect for bacterial infectious disease treatment. EPIs inhibit efflux pump activity leading to inactive drug transport. EPIs do not use the same molecular target as antibiotics and can be used as adjuncts in combination with antibiotics to enhance their activity. Efflux inhibitors have been developed against the efflux pumps having the greatest effect on antibiotic susceptibility,^1^ which in *E. coli* is AcrB. One of the required characteristics of an EPI to make it usable in the clinical setting is to not lead to selection of resistance to its action. A compensatory increased expression of other efflux pumps, which are present in the cells as systems with redundant functions and that have been shown to have an overlapping range of substrates including antibiotics, might be a resistance mechanism to molecules inhibiting AcrAB.

The aim of this work was to investigate the molecular basis and consequences of efflux pump expression and to determine whether exposure to efflux inhibitors confers increased expression of other efflux pumps, which could undermine the development of efflux inhibitors as a drug discovery strategy. Except for *acrB*, basal expression of the other eight efflux pump genes tested was low. This is consistent with data previously described by Sulavik *et al.*^12^ who in 2001 showed that, except for AcrAB, MdfA and EmrE which confer drug resistance when overexpressed, the other pumps are poorly expressed under normal laboratory conditions.

Our data suggests that use of an AcrB (efflux) inhibitor will not lead to compensatory increases in expression of the eight investigated efflux pump genes in *E. coli*. Our data further showed that PAβN and chlorpromazine, which act as competitive substrates for AcrB and so act as inhibitors of antibiotic efflux,^24–26^ also caused no change in the transcriptional activity of the efflux pump genes. PAβN significantly decreased the MICs of chloramphenicol, erythromycin and novobiocin, which are substrates of AcrB, while chlorpromazine had no significant effect, as was observed in a previous study.^24^ Data comparing the activity of erythromycin against the wild-type, Δ*acrB* and AcrB(D408A) strains suggests that erythromycin is a substrate of AcrB. Therefore, these data suggest that erythromycin may be exported by other efflux pumps (which may be inhibited by PAβN), or it could be due to the membrane-permeabilizing effects of PAβN allowing erythromycin, a relatively large antibiotic, to gain better access to the bacterium.^33^

There is conflicting evidence in the literature as to whether expression of other efflux pump genes is increased in response to the deletion of *acrB* in *E. coli.*^11^^,^^34^^,^^35^ As the susceptibility of eight efflux gene loss-of-function mutants was unchanged in our study, this suggests that in *E. coli* efflux pumps other than AcrB do not significantly contribute to inherent drug-resistance to the antibiotics and other antimicrobials and dyes investigated. Wang-Kan *et al.*^23^ showed that in *Salmonella* Typhimurium the *acrB* D408A mutation results in a strain expressing and producing a normal amount of AcrB protein but the protein is functional; in our study, introduction of the *acrB* D408A mutation in *E. coli* MG1655 did not induce overexpression of other RND efflux pumps. Furthermore, except for a 2.6-fold increase in expression of *macB*, no induction was observed when the *acrB* gene was deleted. Overall, our results in *E. coli* MG1655 correlate with data published by Sulavik *et al.*^12^ for *E. coli* W3110 and by Nishino *et al.*^3^ in an *E. coli* TG1 *acrB* deletion mutant.

Data published by Alon Cudkowicz and Schuldiner^36^ and Viveiros *et al.*^37^ for *E. coli* evolved in the presence of AcrB substrates, such as chloramphenicol and tetracycline, showed that efflux pump genes (including *acrF, macB* and *mdfA*) were overexpressed in their *acrB* deletion mutants (BW25113 and AG100, respectively), as a result of efflux pump up-regulation in the absence of the *acrB* gene, suggesting a compensatory role of these pumps in drug efflux. However, this compensation was related to a specific condition, and where the strain had evolved in presence of a pump substrate.

In conclusion, inhibition by chlorpromazine or PAβN, absence, or loss-of-function of *E. coli* AcrB did not significantly increase expression of other efflux pump genes suggesting there is no compensatory mechanism to overcome efflux inhibition. In the conditions tested, the other efflux pumps in *E. coli* provide no contribution to MDR, thus supporting the discovery of inhibitors of AcrAB as antibiotic adjuvants.^38^

## Supplementary Material

dkab452_Supplementary_Data
