# Supplementary material for: Absence, loss-of-function, or inhibition of Escherichia coli AcrB does not increase expression of other efflux pump genes supporting the discovery of AcrB inhibitors as antibiotic adjuvants
Source: J Antimicrob Chemother. 2021 Dec 13;77(3):633–40. doi: 10.1093/jac/dkab452 (PMC8865010; doi:10.1093/jac/dkab452)
Supplement: dkab452_Supplementary_Data [file dkab452_supplementary_data.docx]

**Supplementary data**

**Table S1.** Primers used for constructing efflux pump mutation

| Primer name | Sequence (5’–3’) | Tm (°C) | Gene Synthesis |
| --- | --- | --- | --- |
| *acrD_synth_F* | GGAACCTTCTCCGTACTTTAC | 61.1 | *^a^* |
| *acrD_synth_R* | GATCTGCCCCATCGATTTAC | 61.6 |  |
| *acrF_synth_F* | GGACGTTTGCCATCCTCGCC | *68.6* | *^b^* |
| *acrF_synth_R* | ATTTGCGACATCGATTTTTCCGTCGCTTC | 70.3 |  |
| *mdtB_synth_F* | CACTTTCGCGGTTATGGTGTTTCTCG | 68.4 | *^c^* |
| *mdtB_synth_R* | GCCGATTTCACCTGCGCC | 67.2 |  |
| *macB_synth_F* | GGTGATGAGCAGGTTGAGGTGCT | 69.3 | *^d^* |
| *macB_synth_R* | GGCGTCCAGCGTGGCAAC | 69.4 |  |
| *mdtK_synth_F* | AGCAAATCGCCCTGTTGTACAACG | 68.6 | *^e^* |
| *mdtK_synth_R* | AGCACCCAGTAAGCCGTAAAGG | 67.4 |  |

^a^D408AacrD

GGAACCTTCTCCGTACTTTACGCCTTCGGTTACAGCGTCAACACC

TTAACCATGTTCGCGATGGTGCTGGCGATCGGTCTGCTGGTGGAT

GCCGCCATCGTGGTGGTGGAAAACATCTCAAGAGTGGCAGCGGTT

CTGTTAAGTAACTGAACCCAATGTCGTTAGTGACGCTTACCCGCA

AAAAACCCCGCTTCGGCGGGGTTTTTTCGCTCTTAAGAGGTCACT

GACCTAACAAAAAAAAAACCCCGCCCCTGACAGGGCGGGGTTTTT

TTTGGTCTTGAGTGGCAGAGTCAGTTATCGCGAGCAGTATGTAAG

TAGATCCTCAGTGTCAGCTAGGGATAACAGGGTAATCTGATCCTT

CAACTCAGCAAAAGTTCGATTTATTCAACAAAGCCACGTTGTGTC

TCAAAATCTCTGATGTTACATTGCACAAGATAAAAATATATCATC

ATGAACAATAAAACTGTCTGCTTACATAAACAGTAATACAAGGGG

TGTTATGAGCCATATTCAACGGGAAACGTCTTGCTCGAGGCCGCG

ATTAAATTCCAACATGGATGCTGATTTATATGGGTATAAATGGGC

TCGCGATAATGTCGGGCAATCAGGTGCGACAATCTATCGATTGTA

TGGGAAGCCCGATGCGCCAGAGTTGTTTCTGAAACATGGCAAAGG

TAGCGTTGCCAATGATGTTACAGATGAGATGGTCAGACTAAACTG

GCTGACGGAATTTATGCCTCTTCCGACCATCAAGCATTTTATCCG

TACTCCTGATGATGCATGGTTACTCACCACTGCGATCCCCGGGAA

AACAGCATTCCAGGTATTAGAAGAATATCCTGATTCAGGTGAAAA

TATTGTTGATGCGCTGGCAGTGTTCCTGCGCCGGTTGCATTCGAT

TCCTGTTTGTAATTGTCCTTTTAACAGCGATCGCGTATTTCGTCT

CGCTCAGGCGCAATCACGAATGAATAACGGTTTGGTTGATGCGAG

TGATTTTGATGACGAGCGTAATGGCTGGCCTGTTGAACAAGTCTG

GAAAGAAATGCATAAGCTTTTGCCATTCTCACCGGATTCAGTCGT

CACTCATGGTGATTTCTCACTTGATAACCTTATTTTTGACGAGGG

GAAATTAATAGGTTGTATTGATGTTGGACGAGTCGGAATCGCAGA

CCGATACCAGGATCTTGCCATCCTATGGAACTGCCTCGGTGAGTT

TTCTCCTTCATTACAGAAACGGCTTTTTCAAAAATATGGTATTGA

TAATCCTGATATGAATAAATTGCAGTTTCATTTGATGCTCGATGA

GTTTTTCTAAGCAGGGCGGGGCGTAAGCGATCGGTCTGCTGGTGG

ATGCCGCCATCGTGGTGGTGGAAAACGTCGAACGTATTATGAGTG

AGGAAGGACTCACTCCTCGCGAAGCCACACGTAAATCGATGGGGC

AGATC

^b^D408AacrF

GGACGTTTGCCATCCTCGCCGCTTTTGGTTACTCCATCAA

CACACTAACGATGTTCGGGATGGTGCTTGCCATCGGGCTGCTCGT

CGATGCTGCGATAGTGGTGGTGGAGAACATCTCAAGAGTGGCAGC

GGTTCTGTTAAGTAACTGAACCCAATGTCGTTAGTGACGCTTACC

CGCAAAAAACCCCGCTTCGGCGGGGTTTTTTCGCTCTTAAGAGGT

CACTGACCTAACAAAAAAAAAACCCCGCCCCTGACAGGGCGGGGT

TTTTTTTGGTCTTGAGTGGCAGAGTCAGTTATCGCGAGCAGTATG

TAAGTAGATCCTCAGTGTCAGCTAGGGATAACAGGGTAATCTGAT

CCTTCAACTCAGCAAAAGTTCGATTTATTCAACAAAGCCACGTTG

TGTCTCAAAATCTCTGATGTTACATTGCACAAGATAAAAATATAT

CATCATGAACAATAAAACTGTCTGCTTACATAAACAGTAATACAA

GGGGTGTTATGAGCCATATTCAACGGGAAACGTCTTGCTCGAGGC

CGCGATTAAATTCCAACATGGATGCTGATTTATATGGGTATAAAT

GGGCTCGCGATAATGTCGGGCAATCAGGTGCGACAATCTATCGAT

TGTATGGGAAGCCCGATGCGCCAGAGTTGTTTCTGAAACATGGCA

AAGGTAGCGTTGCCAATGATGTTACAGATGAGATGGTCAGACTAA

ACTGGCTGACGGAATTTATGCCTCTTCCGACCATCAAGCATTTTA

TCCGTACTCCTGATGATGCATGGTTACTCACCACTGCGATCCCCG

GGAAAACAGCATTCCAGGTATTAGAAGAATATCCTGATTCAGGTG

AAAATATTGTTGATGCGCTGGCAGTGTTCCTGCGCCGGTTGCATT

CGATTCCTGTTTGTAATTGTCCTTTTAACAGCGATCGCGTATTTC

GTCTCGCTCAGGCGCAATCACGAATGAATAACGGTTTGGTTGATG

CGAGTGATTTTGATGACGAGCGTAATGGCTGGCCTGTTGAACAAG

TCTGGAAAGAAATGCATAAGCTTTTGCCATTCTCACCGGATTCAG

TCGTCACTCATGGTGATTTCTCACTTGATAACCTTATTTTTGACG

AGGGGAAATTAATAGGTTGTATTGATGTTGGACGAGTCGGAATCG

CAGACCGATACCAGGATCTTGCCATCCTATGGAACTGCCTCGGTG

AGTTTTCTCCTTCATTACAGAAACGGCTTTTTCAAAAATATGGTA

TTGATAATCCTGATATGAATAAATTGCAGTTTCATTTGATGCTCG

ATGAGTTTTTCTAAGCAGGGCGGGGCGTAAGCCATCGGGCTGCTC

GTCGATGCTGCGATAGTGGTGGTGGAGAACGTCGAGCGCGTGATG

ATGGAGGATAAGCTCCCGCCAAAAGAAGCGACGGAAAAATCGATG

TCGCAAAT

^c^D408AmdtB

CACTTTCGCGGTTATGGTGTTTCTCGATTTTTCAATCAAT

AACCTGACACTGATGGCGTTAACTATCGCCACCGGATTCGTGGTC

GATGCGGCCATCGTGGTGATCGAAAACAATCTCAAGAGTGGCAGC

GGTTCTGTTAAGTAACTGAACCCAATGTCGTTAGTGACGCTTACC

CGCAAAAAACCCCGCTTCGGCGGGGTTTTTTCGCTCTTAAGAGGT

CACTGACCTAACAAAAAAAAAACCCCGCCCCTGACAGGGCGGGGT

TTTTTTTGGTCTTGAGTGGCAGAGTCAGTTATCGCGAGCAGTATG

TAAGTAGATCCTCAGTGTCAGCTAGGGATAACAGGGTAATCTGAT

CCTTCAACTCAGCAAAAGTTCGATTTATTCAACAAAGCCACGTTG

TGTCTCAAAATCTCTGATGTTACATTGCACAAGATAAAAATATAT

CATCATGAACAATAAAACTGTCTGCTTACATAAACAGTAATACAA

GGGGTGTTATGAGCCATATTCAACGGGAAACGTCTTGCTCGAGGC

CGCGATTAAATTCCAACATGGATGCTGATTTATATGGGTATAAAT

GGGCTCGCGATAATGTCGGGCAATCAGGTGCGACAATCTATCGAT

TGTATGGGAAGCCCGATGCGCCAGAGTTGTTTCTGAAACATGGCA

AAGGTAGCGTTGCCAATGATGTTACAGATGAGATGGTCAGACTAA

ACTGGCTGACGGAATTTATGCCTCTTCCGACCATCAAGCATTTTA

TCCGTACTCCTGATGATGCATGGTTACTCACCACTGCGATCCCCG

GGAAAACAGCATTCCAGGTATTAGAAGAATATCCTGATTCAGGTG

AAAATATTGTTGATGCGCTGGCAGTGTTCCTGCGCCGGTTGCATT

CGATTCCTGTTTGTAATTGTCCTTTTAACAGCGATCGCGTATTTC

GTCTCGCTCAGGCGCAATCACGAATGAATAACGGTTTGGTTGATG

CGAGTGATTTTGATGACGAGCGTAATGGCTGGCCTGTTGAACAAG

TCTGGAAAGAAATGCATAAGCTTTTGCCATTCTCACCGGATTCAG

TCGTCACTCATGGTGATTTCTCACTTGATAACCTTATTTTTGACG

AGGGGAAATTAATAGGTTGTATTGATGTTGGACGAGTCGGAATCG

CAGACCGATACCAGGATCTTGCCATCCTATGGAACTGCCTCGGTG

AGTTTTCTCCTTCATTACAGAAACGGCTTTTTCAAAAATATGGTA

TTGATAATCCTGATATGAATAAATTGCAGTTTCATTTGATGCTCG

ATGAGTTTTTCTAAGCAGGGCGGGGCGTAACGCCACCGGATTCGT

GGTCGATGCGGCCATCGTGGTGATCGAAAACATTTCCCGCTATAT

CGAAAAAGGCGAAAAACCGTTGGCGGCGGCGCTCAAGGGCGCAGG

TGAAATCGGC

^d^K47LmacB

GGTGATGAGCAGGTTGAGGTGCTGAAGGGCATCAGCCTCG

ATATTTATGCGGGTGAGATGGTCGCGATTGTTGGCGCTTCGGGTT

CCGGTCTGTCGACCCTGATGAATATTCTCGATCTCAAGAGTGGCA

GCGGTTCTGTTAAGTAACTGAACCCAATGTCGTTAGTGACGCTTA

CCCGCAAAAAACCCCGCTTCGGCGGGGTTTTTTCGCTCTTAAGAG

GTCACTGACCTAACAAAAAAAAAACCCCGCCCCTGACAGGGCGGG

GTTTTTTTTGGTCTTGAGTGGCAGAGTCAGTTATCGCGAGCAGTA

TGTAAGTAGATCCTCAGTGTCAGCTAGGGATAACAGGGTAATCTG

ATCCTTCAACTCAGCAAAAGTTCGATTTATTCAACAAAGCCACGT

TGTGTCTCAAAATCTCTGATGTTACATTGCACAAGATAAAAATAT

ATCATCATGAACAATAAAACTGTCTGCTTACATAAACAGTAATAC

AAGGGGTGTTATGAGCCATATTCAACGGGAAACGTCTTGCTCGAG

GCCGCGATTAAATTCCAACATGGATGCTGATTTATATGGGTATAA

ATGGGCTCGCGATAATGTCGGGCAATCAGGTGCGACAATCTATCG

ATTGTATGGGAAGCCCGATGCGCCAGAGTTGTTTCTGAAACATGG

CAAAGGTAGCGTTGCCAATGATGTTACAGATGAGATGGTCAGACT

AAACTGGCTGACGGAATTTATGCCTCTTCCGACCATCAAGCATTT

TATCCGTACTCCTGATGATGCATGGTTACTCACCACTGCGATCCC

CGGGAAAACAGCATTCCAGGTATTAGAAGAATATCCTGATTCAGG

TGAAAATATTGTTGATGCGCTGGCAGTGTTCCTGCGCCGGTTGCA

TTCGATTCCTGTTTGTAATTGTCCTTTTAACAGCGATCGCGTATT

TCGTCTCGCTCAGGCGCAATCACGAATGAATAACGGTTTGGTTGA

TGCGAGTGATTTTGATGACGAGCGTAATGGCTGGCCTGTTGAACA

AGTCTGGAAAGAAATGCATAAGCTTTTGCCATTCTCACCGGATTC

AGTCGTCACTCATGGTGATTTCTCACTTGATAACCTTATTTTTGA

CGAGGGGAAATTAATAGGTTGTATTGATGTTGGACGAGTCGGAAT

CGCAGACCGATACCAGGATCTTGCCATCCTATGGAACTGCCTCGG

TGAGTTTTCTCCTTCATTACAGAAACGGCTTTTTCAAAAATATGG

TATTGATAATCCTGATATGAATAAATTGCAGTTTCATTTGATGCT

CGATGAGTTTTTCTAAGCAGGGCGGGGCGTAATGTTGGCGCTTCG

GGTTCCGGTCTGTCGACCCTGATGAATATTCTCGGCTGTCTGGAT

AAGGCCACCAGCGGCACCTATCGCGTCGCCGGTCAGGATGTTGCC

ACGCTGGACGCC

^e^D368AmdtK

AGCAAATCGCCCTGTTGTACAACGACAATCCCGAGGTTGT

AACGCTGGCTGCGCATTTGATGTTGCTGGCGGCGGTATATCAGAT

TTCTGCCTCAATCCAGGTGATTGGCAGTATCTCAAGAGTGGCAGC

GGTTCTGTTAAGTAACTGAACCCAATGTCGTTAGTGACGCTTACC

CGCAAAAAACCCCGCTTCGGCGGGGTTTTTTCGCTCTTAAGAGGT

CACTGACCTAACAAAAAAAAAACCCCGCCCCTGACAGGGCGGGGT

TTTTTTTGGTCTTGAGTGGCAGAGTCAGTTATCGCGAGCAGTATG

TAAGTAGATCCTCAGTGTCAGCTAGGGATAACAGGGTAATCTGAT

CCTTCAACTCAGCAAAAGTTCGATTTATTCAACAAAGCCACGTTG

TGTCTCAAAATCTCTGATGTTACATTGCACAAGATAAAAATATAT

CATCATGAACAATAAAACTGTCTGCTTACATAAACAGTAATACAA

GGGGTGTTATGAGCCATATTCAACGGGAAACGTCTTGCTCGAGGC

CGCGATTAAATTCCAACATGGATGCTGATTTATATGGGTATAAAT

GGGCTCGCGATAATGTCGGGCAATCAGGTGCGACAATCTATCGAT

TGTATGGGAAGCCCGATGCGCCAGAGTTGTTTCTGAAACATGGCA

AAGGTAGCGTTGCCAATGATGTTACAGATGAGATGGTCAGACTAA

ACTGGCTGACGGAATTTATGCCTCTTCCGACCATCAAGCATTTTA

TCCGTACTCCTGATGATGCATGGTTACTCACCACTGCGATCCCCG

GGAAAACAGCATTCCAGGTATTAGAAGAATATCCTGATTCAGGTG

AAAATATTGTTGATGCGCTGGCAGTGTTCCTGCGCCGGTTGCATT

CGATTCCTGTTTGTAATTGTCCTTTTAACAGCGATCGCGTATTTC

GTCTCGCTCAGGCGCAATCACGAATGAATAACGGTTTGGTTGATG

CGAGTGATTTTGATGACGAGCGTAATGGCTGGCCTGTTGAACAAG

TCTGGAAAGAAATGCATAAGCTTTTGCCATTCTCACCGGATTCAG

TCGTCACTCATGGTGATTTCTCACTTGATAACCTTATTTTTGACG

AGGGGAAATTAATAGGTTGTATTGATGTTGGACGAGTCGGAATCG

CAGACCGATACCAGGATCTTGCCATCCTATGGAACTGCCTCGGTG

AGTTTTCTCCTTCATTACAGAAACGGCTTTTTCAAAAATATGGTA

TTGATAATCCTGATATGAATAAATTGCAGTTTCATTTGATGCTCG

ATGAGTTTTTCTAAGCAGGGCGGGGCGTAAGCGGCGGTATATCAG

ATTTCTGCCTCAATCCAGGTGATTGGCAGTGGGATTTTGCGTGGT

TATAAAGATACGCGTTCCATTTTCTATATTACCTTTACGGCTTAC

TGGGTGCT

**Table S2.** Primers used to validate efflux pump mutation

| Primer name | Sequence (5’–3’) | Tm (°C) | Amplicon length (bp) |
| --- | --- | --- | --- |
| *acrB-408D-checkF* | CCATCGGCCTGTTGGTGGATGA | 72 | 764 |
| *acrB-408A-checkF* | CATCGGCCTGTTGGTGGATGC | 72 |  |
| *acrB-int-checkR* | GAAAGCGCGTGTTGCACGC | 71 |  |
| *acrD-408D-checkF* | CGATCGGTCTGCTGGTGGATGA | 72 | 720 |
| *acrD-408A-checkF* | GATCGGTCTGCTGGTGGATGC | 71 |  |
| *acrD-int-checkR* | CGGTCTTACTGTCGCGTTCGC | 71 |  |
| *acrF-408D-checkF* | CATCGGGCTGCTCGTCGATGA | 72 | 715 |
| *acrF-408A-checkF* | CATCGGGCTGCTCGTCGATGC | 74 |  |
| *acrF-int-checkR* | GTCACCATTACGCTCTTCCCACG | 70 |  |
| *macB-47K-checkF* | GCGCTTCGGGTTCCGGTAAA | 71 | 743 |
| *macB-47L-checkF* | CGCTTCGGGTTCCGGTCTG | 72 |  |
| *macB-int-checkR* | GGAAACCACCGACGCAATACCG | 71 |  |
| *mdtK-368D-checkF* | GCTGGCGGCGGTATATCAGATTTCTGA | 72 | 707 |
| *mdtK-368A-checkF* | TGGCGGCGGTATATCAGATTTCTGC | 71 | 700 |
| *mdtK_upst_R* | TGAGAGACTAAAAAGCCGCCTGAATGG | 65 |  |
| *acrB upst F* | CTG ATC ACC AGT GAC GGC AT | 59.4 | 600 |
| *acrB downst R* | CGT ATG AGA TCC TGA GTT GG | 57.3 |  |

Primers were designed based on the sequence for the *E. coli* K-12 complete genome (accession number NC_000913).

**Table S3.** Primers used for constructing efflux transcriptional GFP reporters

| Primer name | Sequence (5’–3’) | Tm (°C) | Description |
| --- | --- | --- | --- |
| *BamHI-PacrA-Fw* | aaaaaaggatCCACATCGAGGATGTGTTGG | 64 | Anneals 199-180 bp upstream of acrA start codon, amplifies towards acrA, with the BamHI restriction site |
| *XbaI-PacrA-Rv* | aaaaaatctagaCCGATTTCAAATTGGTCAATGGTC | 64 | Anneals 18-41 bp upstream of acrA start codon, amplifies away from acrA, with XbaI restriction site |
| *BamHI-PacrD-Fw* | aaaaaaggatCCAAACTGACCCTCGACTG | 63 | Anneals 519-501 bp upstream of acrD start codon, amplifies towards acrD, with the BamHI restriction site |
| *XbaI-PacrD-Rv* | aaaaaatctagaGCGTATCGTTGTAGGCTTGAC | 64 | Anneals 23-43 bp upstream of acrD start codon, amplifies away from acrD, with XbaI restriction site |
| *BamHI-PacrE-Fw* | aaaaaaggatccGAAGGCTGTTGCAACCAC | 63 | Anneals 601-584 bp upstream of acrE start codon, amplifies towards acrE, with the BamHI restriction site |
| *XbaI-PacrE-Rv* | aaaaaatctagAAAAGCGCGTTATTTACCCA | 62 | Anneals 20-39 bp upstream of acrE start codon, amplifies away from acrE, with XbaI restriction site |
| *BamHI-PemrA-Fw* | cacataggatcCGCCCATTGAACAAATGC | 61 | Anneals 641-624 bp upstream of emrA start codon, amplifies towards emrA, with the BamHI restriction site |
| *XbaI-PemrA-Rv* | ctcatatctagaCGATCTTCTTAACTCATCGGC | 62 | Anneals 11-32 bp upstream of emrA start codon, amplifies away from emrA, with XbaI restriction site |
| *BamHI-PmacA-Fw* | aaaaaaggatcCGTCAGTGCGGACATCA | 65 | Anneals 1571-1555 bp upstream of macA start codon, amplifies towards macA, with the BamHI restriction site. |
| *XbaI-PmacA-Rv* | aaaaaatctagATGCCAATCCGGGTCAT | 64 | Anneals 21-37 bp upstream of macA start codon, amplifies away from macA, with XbaI restriction site |
| *BamHI-PcusC-Fw* | aaaaacggatcCTGCGAGGCGATAAGCG | 64 | Anneals 690-674 bp upstream of cusC start codon, amplifies towards cusC, with the BamHI restriction site. |
| *XbaI-PcusC-Rv* | cccaactctagaGGTGATTTTATGCCGCCAAC | 63 | Anneals 16-35 bp upstream of cusC start codon, amplifies away from cusC, with XbaI restriction site |
| *BamHI-PmdfA-Fw* | aaaaaaggatcCTTCCCGACGCAAAGT | 62 | Anneals 282-267 bp upstream of mdfA start codon, amplifies towards mdfA, with the BamHI restriction site. |
| *XbaI-PmdfA-Rv* | aaaaaatctagAATAATAATCGCGCAGAGT | 58 | Anneals 15-33 bp upstream of mdfA start codon, amplifies away from mdfA, with XbaI restriction site |
| *BamHI-PmdtA-Fw* | cacataggatccGTCTATCGCTAACGCGTAGA | 62 | Anneals 181-162 bp upstream of mdtA start codon, amplifies towards mdtA, with the BamHI restriction site. |
| *XbaI-PmdtA-Rv* | aaaaaatctaGAAACGTTTCGCGGAATGA | 62 | Anneals 21-39 bp upstream of mdtA start codon, amplifies away from mdtA, with XbaI restriction site |
| *BamHI-PmdtK-Fw* | aaaaaaggatcCGCCACTTCAGCAGTGGTC | 69 | Anneals 544-525 bp upstream of mdtK start codon, amplifies towards mdtK, with the BamHI restriction site. |
| *XbaI-PmdtK-Rv* | aaaaaatctaGAGAGACTAAAAAGCCGCCTGA | 69 | Anneals 28-50 bp upstream of mdtK start codon, amplifies away from mdtK, with XbaI restriction site |
| pMW82 *BamHI-Rv* | tttttGGATCCTCTACGCCG | 62 | Amplify the whole pMW82 to allow cloning of promoters using the XbaI/BamHI |
| pMW82 *XbaI-Fw* | ttttttTCTAGATTTAAGAAGGAGATATACATATG | 59 |  |

Primers were designed based on the sequence for the *E. coli* K-12 complete genome (accession number NC_000913).

Table S4. Inducing conditions for efflux pumps gene expression

| EP | Inducer described in literature | Induction level | Method | Reference |
| --- | --- | --- | --- | --- |
| *acrAB* | *tolC* deletion | 2.3x | qPCR | Ruiz and Levy, 2014 |
| *acrD* | ZnSO_4_  ZnSO_4_  Overexpression of EvgA/BaeR/CpxR/OmpR | 17x  6.2x  1.7/15/4.1/10x | qPCR  Microarray  qPCR | Wang & Fierke, 2013  Lee et al., 2005  Hirakawa et al., 2003 |
| *acrEF* | Overexpression of CpxR/OmpR | 1.4/3.3x | qPCR | Hirakawa et al., 2003 |
| *cusCFBA* | ZnSO_4_  CuSO_4_ | 11x  from 100 to 800 units | Microarray  β-gal reporter | Lee et al., 2005  Munson et al., 2000 |
| *emrAB* | Overexpression of OmpR/CpxR/EvgA  Constitutive activation of EvgS | 6.5/2.5/2.0x  1.2x | qPCR  Microarray | Hirakawa et al., 2003  Eguchi et al., 2003 |
| *macAB* | Overexpression of OmpR | 3.1x | qPCR | Hirakawa et al., 2003 |
| *mdfA* | Constitutive activation of EvgS  Overexpression of OmpR | 4.4x  3.1x | Microarray  qPCR | Eguchi et al., 2003  Hirakawa et al., 2003 |
| *mdtABC* | ZnSO_4_  ZnSO_4_  Overexpression of BaeR | 7x  2.1x  7x | qPCR  Microarray  qPCR | Wang & Fierke, 2013  Lee et al., 2005  Nagakubo et al., 2002 |
| *mdtK* | Overexpression of OmpR | 3.2x | qPCR | Hirakawa et al., 2003 |

**Table S5.** Primers used for qPCR on transcriptional GFP reporters

| Primer name | Sequence (5’–3’) | Tm (°C) | | Amplicon length (bp) | |
| --- | --- | --- | --- | --- | --- |
| *acrA qPCR Fw* | AAGCAGGTGTCTCTCTCTAT | | 55.3 | | 141 |
| *acrA qPCR Rv* | TACCGAGCAGTTTCTGATAA | | 53.2 | |  |
| *acrD qPCR Fw* | TGGAATCGTTAGTGAAGCAG | | 56.3 | | 138 |
| *acrD qPCR Rv* | CAGCCAGACACAGGAATAC | | 56.7 | |  |
| *acrE qPCR Fw* | CCATTCTTATCCCGCAACAA | | 55.3 | | 131 |
| *acrE qPCR Rv* | CAACCATTTATCGCCAATCG | | 55.3 | |  |
| *cusC qPCR Fw* | GTCTCCTTGTAAACTTCTGC | | 55.3 | | 120 |
| *cusC qPCR Rv* | CCGTTCTGGCTGAGTGAG | | 58.2 | |  |
| *macA qPCR Fw* | CGGAGATGGCTGTGAAAC | | 56.0 | | 144 |
| *macA qPCR Rv* | TGGTGATTTGCGTGACTTC | | 54.5 | |  |
| *mdfA qPCR Fw* | TTGATTGGGTTCCTACTTCG | | 55.3 | | 141 |
| *mdfA qPCR Rv* | CCAGACAGGTGACGATAAAC | | 57.3 | |  |
| *mdtA qPCR Fw* | GAAACCGAAGGCACCATTA | | 54.5 | | 125 |
| *mdtA qPCR Rv* | TTACCAACATCAACCTGCTT | | 53.2 | |  |
| *mdtK qPCR Fw* | TAATGTTCGTGCTTCCAATG | | 53.2 | | 128 |
| *mdtK qPCR Rv* | CATACAGACACCCACCATAA | | 55.3 | |  |
| *pMW82 gfp qPCR Fw* | AGAGGGTGAAGGTGATGC | | 55.6 | | 136 |
| *pMW82 gfp qPCR Rv* | ATCTGGGTATCTCGCAAAGC | | 57.3 | |  |
| *16S rRNA qPCR Fw* | GCTAATACCGCATAACGTCG | | 57.3 | | 139 |
| *16S rRNA qPCR Rv* | TCATCCTCTCAGACCAGCTA | | 57.3 | |  |

Primers were designed based on the sequence for the *E. coli* K-12 complete genome (accession number NC_000913).

Table S6. Relative expression of efflux pump genes and *gfp* under control of the efflux pump gene regulatory region.

|  | Copies of transcript per copy of 16S rRNA | |  |
| --- | --- | --- | --- |
| Regulatory region | Reported gene | *gfp* | Ratio *gfp* : reported gene |
| *acrAB* | 0.074 | 0.277 | 3.7 |
| *acrD* | 0.025 | 0.122 | 5.0 |
| *acrEF* | 0.012 | 0.054 | 4.5 |
| *cusCFBA* | 0.014 | 0.073 | 4.2 |
| *macAB* | 0.038 | 0.161 | 5.1 |
| *mdfA* | 0.033 | 0.255 | 7.7 |
| *mdtABC* | 0.023 | 0.152 | 6.7 |
| *mdtK* | 0.041 | 0.116 | 2.8 |

**Figure S1.** Fold change in GFP fluorescence in *E.coli* BW25113 Δ*tolC* mutant background. Values are averages of two biological and three technical replicates for each reporter strain. Maximum specific fluorescence was seen at OD_600nm_ 0.6. Student's t-test was performed comparing the maximum fluorescence value achieved in *tolC* deletion mutant with the fluorescence value of the corresponding WT strain; values of *P <*0.05 indicate significance.

ev, empty vector.

*****

*****

**Figure S2.** Fold change in GFP fluorescence in *E.coli* MG1655 in the presence of 0.5 mM CuSO4 compared to no-compound condition. Values are averages of two biological and three technical replicates for each reporter strain. Maximum specific fluorescence was seen at OD_600nm_ 0.6. Student's t-test was performed comparing the maximum fluorescence value achieved in the presence of compound with the fluorescence value of the culture in its absence; with values of P < 0.05 indicate significance. ev, empty vector

*****

**GFP assays to confirm reporter construct activity**

Reporter plasmid functionality was tested by ensuring that they respond to conditions that are reported to alter expression of the genes upon which they report (Supplementary Table 4).

**Methods**

Except for the experiments to determine the effect of pH, MOPS minimal medium (Teknova) supplemented with glucose, 400 mg/L histidine and 1 mg/L thiamine was used throughout these assays. To determine the effect of pH, M9 medium was prepared with the pH buffering range adjusted by varying the ratio of dibasic and monobasic potassium phosphate: for pH 5.8, 4 mM K_2_HPO_4_ and 62 mM KH_2_PO_4_, for pH7.3, 40 mM K_2_HPO_4_ and 22 mM KH_2_PO_4_ and for pH 8.1, 62 mM K_2_HPO_4_ and 4 mM KH_2_PO_4_. For experiments testing chemical induction, 10 μL of test substances at 10x final concentration were used. Overnight cultures were diluted into fresh medium, incubated at 37°C until OD_600_ of approximately 0.6 was reached and 90 μL per well used to inoculate black-sided, clear flat-bottomed 96-well plates. GFP fluorescence (excitation 492 nm, emission 520 nm) and OD_600_ were measured every 3 minutes for 15 hours on a FLUOstar Omega plate reader (BMG Labtech).

For experiments measuring the GFP expression level in *acrB* or *tolC* or *EvgS* mutant strains, overnight cultures were diluted 1/1000 and 100 μL per well used to inoculate assay plates. The blank corrected fluorescence was divided by the OD600 to give specific fluorescence (units of fluorescence per unit OD). The maximum specific fluorescence at any time-point was used for comparative purpose.

**Results**

In a comprehensive study by Hirakawa et al. (Hirakawa,H. 2003) overexpression of *ompR* caused increased expression of *acrE* (3.3-fold), *emrA* (6.5-fold), *mdfA* (3.1-fold), *macA* (3.1-fold), *mdtK* (*ydhE*, 3.2-fold), and an approximately 50% decrease in *acrA* expression. As the EnvZ/OmpR system is activated by K+ (Jung,K. 2001) we hypothesised that addition of KCl would have an effect on expression of these efflux pump genes. However, when 4 M KCl was added no induction was observed.

Being the *emrAB* and *mdfA* genes regulated by the pH-responsive two-component system EvgAS (Eguchi,Y. 2003), the effect of pH upon GFP expression was tested in M9-based medium, in which the pH was adjusted by altering the ratio of monobasic and dibasic potassium phosphate. GFP fluorescence was not significantly increased by acidification of the medium for any of the reporter plasmids. Increasing the pH to mildly alkaline conditions (pH 8.1) either had no effect on GFP fluorescence with any construct.

To bypass the acid-sensitive nature of GFP fluorescence, a strain that produces a constitutively active EvgS protein (EvgS S600I mutant, from Johnson, MD. 2014 ) was used as a genetic background for reporter plasmids. When compared to the wild-type strain with the same reporter plasmids, the mutation in *evgS* caused no change in GFP expression from any of the plasmids.

On the other hand, using *E.coli* BW25113 *ΔtolC* as a background strain, GFP expression increased to 2.8-fold with the *acrA* promoter and to 9.6-fold with the *acrE* promoter; the specific fluorescence values were the same for both plasmids in this strain (Supplementary Figure 1).

Both *acrD* and *mdtABC* are regulated by the BaeSR two-component system, which responds to the presence of zinc (Hirakawa,H. 2003; Wang,D. 2013). Meanwhile, expression of *cusCFBA* is regulated by the copper-responsive CusSR two-component system and its expression has also been reported to be increased by the addition of zinc (Lee,LJ. 2005). In our assay, addition of 0.5 mM ZnSO4 did not increase GFP fluorescence with none of these genes promoter. On the other hand, the *cusCFBA* regulatory region responded to the addition of 0.5 mM CuSO4 (Munson,GP. 2000) increasing GFP fluorescence to 17.6-fold the basal level (Supplementary Figure 2).

**References**

1. Ruiz C, Levy SB. Regulation of *acrAB* expression by cellular metabolites in *Escherichia coli.* *J Antimicrob Chemother* 2014; **69**: 390-9.

2. Wang D, Fierke CA. The BaeSR regulon is involved in defense against zinc toxicity in *E. coli.* *Metallomics* 2013; **5**: 372-83.

3. Lee LJ, Barrett JA, Poole RK. Genome-wide transcriptional response of chemostat-cultured *Escherichia coli* to zinc. *J Bacteriol* 2005; **187**: 1124-34.

4. Hirakawa H, Nishino K, Hirata T et al. Comprehensive studies of drug resistance mediated by overexpression of response regulators of two-component signal transduction systems in *Escherichia coli*. *J Bacteriol* 2003; **185**: 1851-6.

5. Munson GP, Lam DL, Outten FW et al. Identification of a copper-responsive two-component system on the chromosome of *Escherichia coli* K-12. *J Bacteriol* 2000; **182**: 5864-71.

6. Eguchi Y, Oshima T, Mori H et al. Transcriptional regulation of drug efflux genes by EvgAS, a two-component system in *Escherichia coli*. *Microbiology* 2003; **149**: 2819-28.

7. Nagakubo S, Nishino K, Hirata T, Yamaguchi A. The putative response regulator BaeR stimulates multidrug resistance of *Escherichia coli* via a novel multidrug exporter system, MdtABC. *J Bacteriol* 2002; Aug;184(15):4161-7.

8. Johnson MD, Bell J, Clarke K et al. Characterization of mutations in the PAS domain of the EvgS sensor kinase selected by laboratory evolution for acid resistance in *Escherichia coli*. *Mol Microbiol* 2014 Sep; 93(5): 911–927.

9. K Jung, K Hamann and A Revermann. K+ stimulates specifically the autokinase activity of purified and reconstituted EnvZ of *Escherichia coli*. *J Biol Chem* . 2001 Nov 2;276(44):40896-9.
